# Public perceptions of health and economic impacts during COVID-19: Findings from a repeated cross-national survey in the US, UK and Germany (2020–2022)

**DOI:** 10.1136/bmjph-2024-001095

**Published:** 2025-10-05

**Authors:** Julia E Koller, Karoline Villinger, Emma Erhard, Harald T Schupp, Britta Renner

**Affiliations:** 1 Department of Psychology, University of Konstanz, Konstanz, Baden-Württemberg, Germany; 2 University of Zurich, Zurich, Switzerland

**Keywords:** COVID-19, Public Health, SARS-CoV-2

## Abstract

**Introduction:**

The COVID-19 pandemic has significantly affected both health and economic domains at individual, national and global levels, sparking public debate about the severity of these impacts and the necessity of protective measures. This study compared the perceived severity of the pandemic’s impacts across three dimensions: (a) domain (health vs economy), (b) target (self, own country, world) and (c) country (US, UK and Germany), over time (April 2020 to January 2022).

**Methods:**

We administered a serial cross-sectional online survey as part of the ‘EUCLID’ project. A total of 78 498 participants from the US, UK and Germany completed the questionnaire across 26 waves per country—78 survey waves in total. Data collection used quota-sampling to approximate national distributions by age group x gender.

**Results:**

Perceived health (M=3.41, SD=1.07) and economic (M=3.69, SD=1.09) impacts were rated as moderate to serious (scale: 1–5). Most participants showed individual optimism, perceiving the impact on themselves as less severe than on their country or the world (e.g., health: 55.6% self vs country, 66.0% self vs world; economy: 66.0% and 67.5%, respectively). Conversely, national optimism was less common (health: 33.5%, economy: 15.9%). These patterns remained largely consistent across countries and time points.

**Conclusion:**

Although the pandemic was fundamentally a health crisis, participants perceived its economic impact as more severe. Most individuals were personally optimistic but showed little national-level optimism. This indicates that they perceived their country to be affected at least as much as, or possibly more than, the global average. Notably, participants in three of the world’s largest economies viewed their national economic situation as equally impacted as the global average—potentially weakening the perceived need for international support and solidarity.

**WHAT IS ALREADY KNOWN ON THIS TOPIC**

- Research on the COVID-19 pandemic has shown that individuals often perceive themselves to be at lower risk than others (individual optimism) and tend to view the health impacts of the pandemic as less severe than its economic impacts.

**WHAT THIS STUDY ADDS**

- This large-scale study (N=78 498) from the US, UK and Germany introduces a socio-spatial perspective, comparing perceived health and economic impacts of the pandemic across three levels: the self, one’s own country and the world. It integrates individual and collective perceptions to explore how people evaluate impacts at multiple scales.

**HOW THIS STUDY MIGHT AFFECT RESEARCH, PRACTICE OR POLICY**

- With regard to future pandemic preparedness, the finding that economic impacts were perceived as more severe than health impacts may have implications for public support of collective protection measures.
- The perception that national economies were equally affected as the global average may contribute to an underestimation of global disparities in mitigation resources, potentially weakening support for international aid and global solidarity.

## Introduction

 The COVID-19 pandemic had an immense impact on individuals and societies worldwide.^1^ Although it began as a health crisis, its impact extended into other domains—particularly the economy—resulting in profound and lasting disruptions.^2^ These consequences were widely debated in the public sphere, as controversy emerged around the perceived trade-offs between economic impacts (e.g., job security, business closures) and public health (e.g., infection control, healthcare system capacity) and the cost–benefit balance of collective protection measures.^3 4^

Governments’ considerations of health and economic risks and impacts varied significantly across countries. This contributed to diverse national responses throughout the pandemic.^5^ In the early stages, many governments imposed strict collective protection measures, either nationwide (e.g., Germany, the United Kingdom (UK)) or regionally (e.g., the United States (US)). However, as the pandemic progressed, some countries shifted more rapidly towards strategies emphasising individual responsibility and voluntary guidelines.

Substantial differences in national health and economic systems also shaped how countries experienced the impacts of the COVID-19 pandemic.^6^ For instance, the UK operates a National Health Service, in which regulation, financing and care provision are managed by the state. In contrast, Germany uses a Social Health Insurance model, where societal actors play a central role in regulation, and the US relies on a predominantly private healthcare system governed largely by market forces.^7^ These structural differences contributed to varying levels of vulnerability, with the pandemic exacerbating pre-existing inequalities in health access and economic stability both within and across countries.^8 9^ As a result, the pandemic’s impact was marked by differences in severity at every scale, from individuals to nations to the global level.

However, how seriously these impacts were perceived for different socio-spatial targets—such as the self, one’s own country or the world—remains underexplored. Two lines of research are particularly relevant here. First, extensive literature on comparative or unrealistic optimism shows that individuals often perceive themselves to be at lower risk than others,^10^,^12^ also in the context of the COVID-19 pandemic.^13 14^ Second, research on spatial optimism finds that people tend to view proximal targets (e.g., themselves or their local area) more favourably than more distant or abstract targets (e.g., their country or the world).^15^,^18^

**Figure 1:**
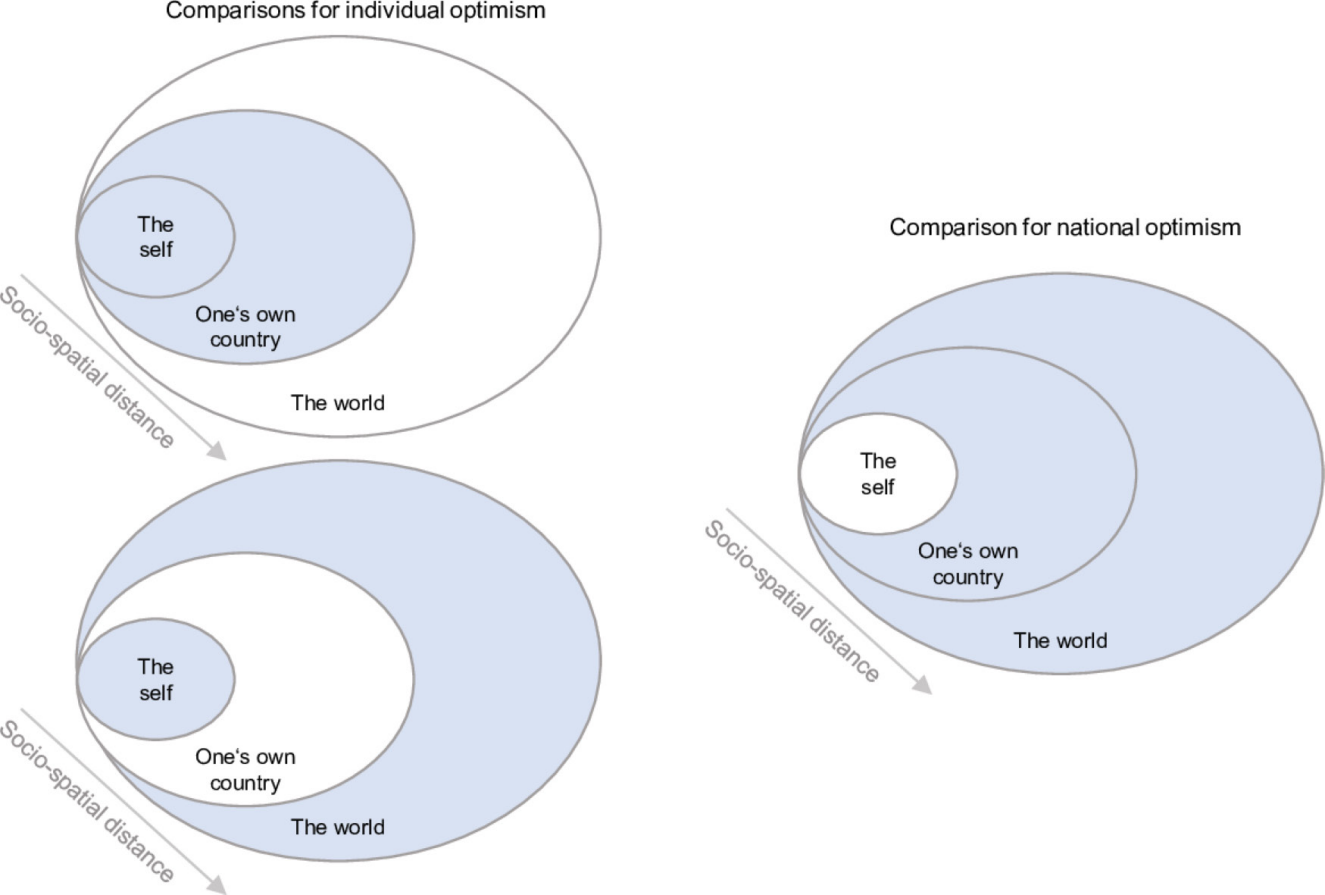
Conceptual framework for assessing individual and national optimism in perceived health and economic impacts across socio-spatial scales (self, country, world). Left panel: Comparisons between perceived individual impacts and national/global impacts, used to assess individual optimism. Right panel: Comparison between national and global impacts, used to assess national optimism. Blue highlights the socio-spatial targets being compared.

Drawing on this literature, we propose that the impact of the COVID-19 pandemic may have been perceived as less severe at the individual level and more serious at national or global levels, reflecting a pattern of individual optimism (see figure 1). Similarly, national optimism may also occur, with the national impact being viewed as less serious than the global impact.

Research on the COVID-19 pandemic suggests that people were more concerned about economic risks than health risks.^14 19 20^ However, it remains unclear whether individuals perceived the severity of actual health and economic impacts differently, especially when considering the socio-spatial target (i.e., self, one’s own country or the world).

In the present study, we investigate how people evaluated the severity of the COVID-19 pandemic’s impact across three key dimensions: (a) domain (health vs economy), (b) socio-spatial target (self, own country, world) and (c) national context (US, UK and Germany), across the period from April 2020 to January 2022. This allows us to explore both the perceived severity of pandemic impacts and observe patterns that can indicate individual optimism (i.e., viewing personal impacts as less severe than national or global ones) as well as national optimism (i.e., viewing national impacts as less severe than global impacts).

## Methods

This study is part of the ‘EUCLID’ project,^21^ which tracked risk perception, protective behaviour and future expectations across multiple countries during the COVID-19 pandemic. In addition to the guidelines of the German Psychological Society and the Declaration of Helsinki, we also adhered to the data security and privacy regulations of the panel providers involved in the sampling process.

### Samples and data

**Figure 2:**
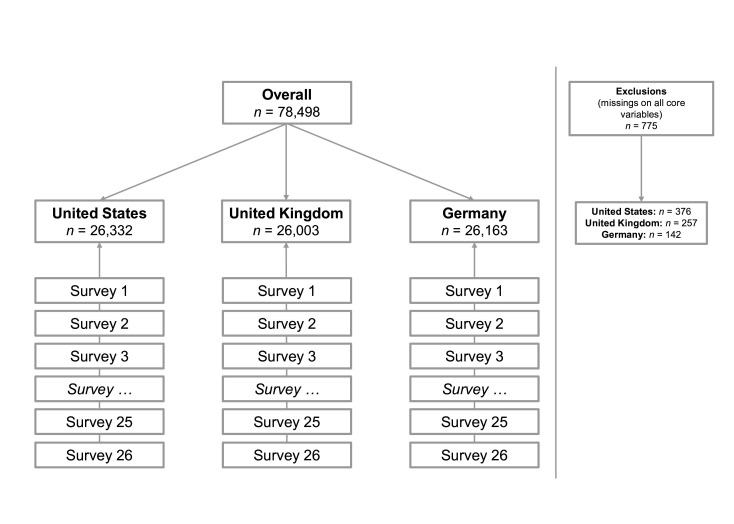
Overview of study design and sampling procedure. For clarity of presentation, not all of the 26 samples collected in each country are shown.

Between 3 April 2020 and 28 January 2022, we administered a serial cross-sectional online survey to 26 non-probability quota samples per country (see figure 2; total N = 78 498, M_age_=46.47, SD=15.86 years; 51.1% women). Each sample consisted of approximately 1 000 participants from the US (n=26 332, M_age_=45.26, SD=16.12 years; 50.8% women), the UK (n = 26 003, M_age_=45.71, SD=15.62 years; 51.3% women) and Germany (n=26 163, M_age_=48.43, SD= 15.63 years; 51.1% women). Samples were quota-based and approximately representative of the respective national populations by gender x five age groups.

Most participants reported good or very good subjective health (67.1%). Additionally, the majority were (self-)employed (61.8%) and reported a monthly net household income of up to 3000 in their respective national currency (56.6%). Educational background was not assessed and is acknowledged as a limitation in the Discussion. For an overview of the sample characteristics for each country and time point, please see online supplemental tables S1-S3.

Using the R package *splitstackshape*,^22^ the first five German samples were drawn from a larger dataset collected via the university study pool, Clickworker,^23^ Prolific Academic^24^ and Facebook advertisements. The remaining German samples, as well as all UK and US samples, were recruited via Splendid Research^25^ and Prolific Academic. Participants were compensated either through the respective platform or via entry into a prize lottery. Attention checks were included to ensure data quality. Cases with missing values on all core variables (i.e., perceived severity of the COVID-19 pandemic’s impact) were excluded from analysis (n = 775).

### Measures of variables

*Perceived severity of the COVID-19 pandemic’s impact*. Participants rated the severity of the pandemic’s health and economic impacts for three socio-spatial targets: the self, their own country and the world. Importantly, our study focuses on *subjective* perceptions rather than objective outcomes. While distinguishing between physical and mental health is conceptually valuable, we adopted a unified framing of ‘health’ to better capture how individuals intuitively assess health threats in real-world contexts. To ensure comparability across domains, we used the same 5-point response scale and socio-spatial structure for both health and economic impact assessments. This symmetry was central to our research aims, enabling direct comparisons of how individuals perceived these two critical domains. Responses were recorded on a scale ranging from (1) ‘not at all serious (negligible)’ to (5) ‘very serious (existential threat)’ (see online supplemental figure S1 or Renner *et al*^26^). We refer to these ratings as *perceptions of absolute impact*, as each rating was analysed independently and not in relation to other responses.

*Optimism*. In line with previous definitions, we defined optimism as a comparatively more favourable perception of the pandemic’s impact for either the self (*individual optimism*) or one’s own country (*national optimism*) relative to more distant socio-spatial targets (e.g., the world).^10 16 18 27^ We applied the indirect method to measure optimism—that is, comparing severity ratings across socio-spatial targets using identical scales rather than asking directly for a comparative judgement.^11 12 28 29^ Difference scores between socio-spatial targets within the same domain (e.g., health) ranged from −4 (pessimism) to +4 (optimism). We refer to these results as perceptions of *relative impact*, in contrast to the *absolute impact* ratings described above.

*New SARS-CoV-2 cases per million people (7-day smoothed*). To account for the real-time context of each survey, we included a country-specific and time-specific indicator of the epidemiological COVID-19 situation: the number of new SARS-CoV-2 cases per million people (7-day rolling average). Data were retrieved from the *Our World in Data* repository (compiled by Edouard Mathieu, Hannah Ritchie, Lucas Rodés-Guirao, Cameron Appel, Daniel Gavrilov, Charlie Giattino, Joe Hasell, Bobbie Macdonald, Saloni Dattani, Diana Beltekian, Esteban Ortiz-Ospina, Max Roser,^30^ based on data from Johns Hopkins University).^31^

### Data analysis procedure

Data were first analysed with visualisations and descriptive statistics (percentages, means and SD) to summarise participants’ perceptions of the severity of health and economic impacts as well as distributions across countries, time points and socio-spatial levels (self, country, world). Subsequently, we employed multilevel modelling^32^ to analyse perceived impacts while accounting for repeated measures—specifically, six items assessing the perceived impact of the COVID-19 pandemic completed by the participants. Multilevel models offer key advantages over repeated-measures analyses (eg, Analysis of Variance), such as accommodating incomplete data and providing comprehensive insights into interindividual variability. All multilevel analyses were conducted in R^33^ using the packages *lme4*^34^ and *lmerTest*.^35^

We analysed both absolute and relative perceptions of impact. In the first step, we analysed the *absolute impact*—that is, the perceived severity of the pandemic’s impact across different socio-spatial targets (self, country, world) and domains (health, economy). Here, perceived severity ratings served as the dependent variable. We modelled differences by domain and target, with the economy and the self used as reference groups. In the second step, we analysed the *relative impact* by analysing difference scores between all socio-spatial targets within the same domain (e.g., self vs country, self vs world, country vs world). This allowed us to directly compare individual optimism (self–country, self–world comparisons) and national optimism (country–world comparison). In these models, the national optimism comparison (country vs world) served as the reference group. As in the absolute impact analyses, we also modelled differences by domain, using the economy as the reference group.

To account for epidemiological context, we included the number of new SARS-CoV-2 cases per million (7-day smoothed) and its interactions with other predictors in an extension of the overall models. Beyond the overall models, we explored variation by country (US, UK, Germany) with separate country-specific models. Furthermore, we calculated additional analyses to control the overall models for age, self-reported health, household income (up to vs above 3000) and employment status, which are reported in online supplemental tables S6 and S7.

For both overall and country-specific analyses, we followed the same model-building strategy. We first fit models with random intercepts to account for individual level differences. Next, we added random slopes for the domain predictor, allowing for individual differences in the effect of domain (health vs economy) on perceived impact. Given the large sample size, we did not rely solely on significance testing (e.g., deviance tests). Instead, model selection was additionally guided by substantive improvements in pseudo-R².^36^ All final models included random slopes for the domain, as they yielded consistent improvements.

## Results

### Perceived health and economic impacts of the COVID-19 pandemic

**Figure 3:**
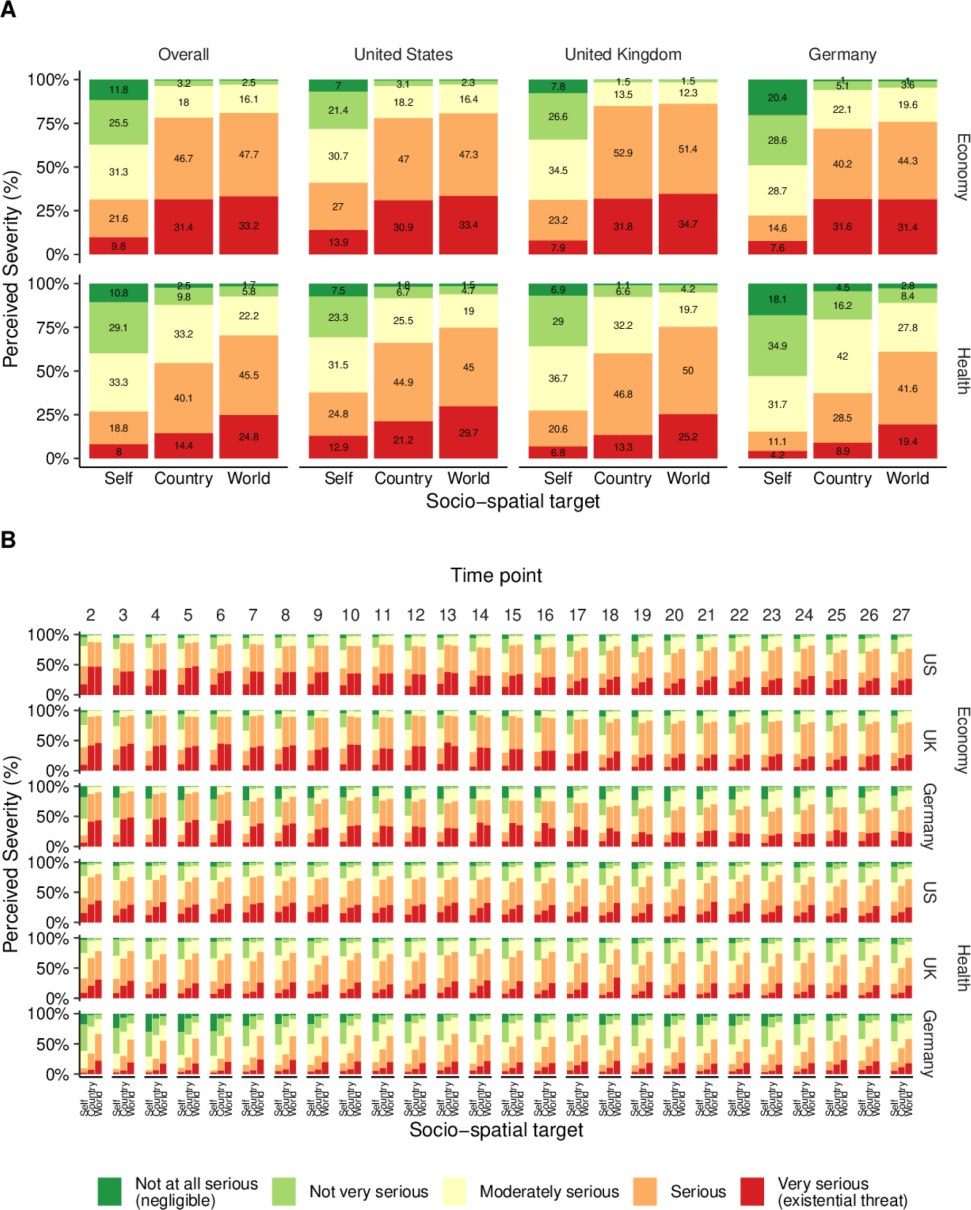
Perceived severity of the COVID-19 pandemic’s health and economic impacts across socio-spatial targets, countries and time point. (A) Proportion of severity ratings by socio-spatial target (self, country, world), domain (health, economy), and country (US, UK, Germany). (B) Proportion of severity ratings by target, domain, country, and time point.

Overall, 80.8% of participants perceived the global economic impact of the COVID-19 pandemic as serious or very serious (M=4.10, SD=0.80 on a scale from 1 to 5; see figure 3A, upper left panel). Similarly, 78.2% rated the impact on their national economy as (very) serious (M=4.05, SD=0.82). In contrast, fewer than one-third (31.4%) perceived the impact on their personal economic situation as serious or very serious (M=2.92, SD=1.15).

Aside from level differences in the perceived severity, a similar pattern emerged across all three countries. In the US sample, the vast majority of participants perceived the global (80.7%, M=4.10, SD=0.80) and national (77.9%, M=4.04, SD=0.82) economic impacts as (very) serious, while only 40.9% considered their personal economic situation to be similarly affected (M=3.19, SD=1.13). In the UK, most participants rated both the global (86.0%, M=4.19, SD=0.72) and national (84.8%, M=4.15, SD=0.71) economic impacts as (very) serious, yet only about one-third (31.1%) considered their personal economic impact as (very) serious (M=2.97, SD=1.06). In Germany, perceptions were slightly more optimistic overall: 75.8% and 71.8% of respondents rated the global (M=4.01, SD=0.86) and national (M=3.96, SD=0.91) economic impacts as (very) serious, while fewer than one-quarter (22.2%) considered their personal economic situation to be seriously affected (M=2.60, SD=1.18).

A similar overall pattern emerged across all three countries over time, despite some differences in the level (see figure 3B and online supplemental table S2). Across time points and countries, the proportion of participants who perceived the personal economic impact as (very) serious was consistently lower than for national and global economic impacts. While a majority generally felt that their country was equally affected as the world, there were several time points in each country when most respondents perceived the national economic impact as more severe than the global one.

Regarding health, 70.3% of participants rated the global health impact of the COVID-19 pandemic as serious or very serious (M=3.86, SD=0.91; see figure 3A, lower left panel). In comparison, 54.5% viewed the national health impact as (very) serious (M=3.54, SD=0.94), while only 26.8% perceived the personal health impact as (very) serious (M=2.84, SD=1.10).

This pattern was largely consistent across countries, again with some variation in perceived severity levels. In the US, 74.8% and 66.0% of participants perceived the global (M=3.97, SD=0.90) and national (M=3.77, SD=0.92) health impacts as (very) serious, while only 37.8% viewed their personal health impact as similarly serious (M=3.12, SD=1.13). In the UK, 75.2% rated the global (M=3.94, SD=0.83) and 60.1% the national health impacts (M=3.65, SD=0.83) as (very) serious, whereas only 27.4% perceived their personal health as seriously affected (M=2.91, SD=1.02). In Germany, ratings were again more optimistic overall: 61.1% and 37.3% considered the global (M=3.67, SD=0.97) and national (M=3.21, SD=0.97) health impacts as (very) serious, but only 15.3% perceived the person health impact as similarly serious (M=2.48, SD=1.04).

Again, a similar pattern was observed across all three countries and the 26 time points, with only minor differences in the perceived severity level (see figure 3B and online supplemental table S2). In all cases, the perceived health impact of the pandemic was consistently rated lowest for the individual and highest at the global level.

**Figure 4:**
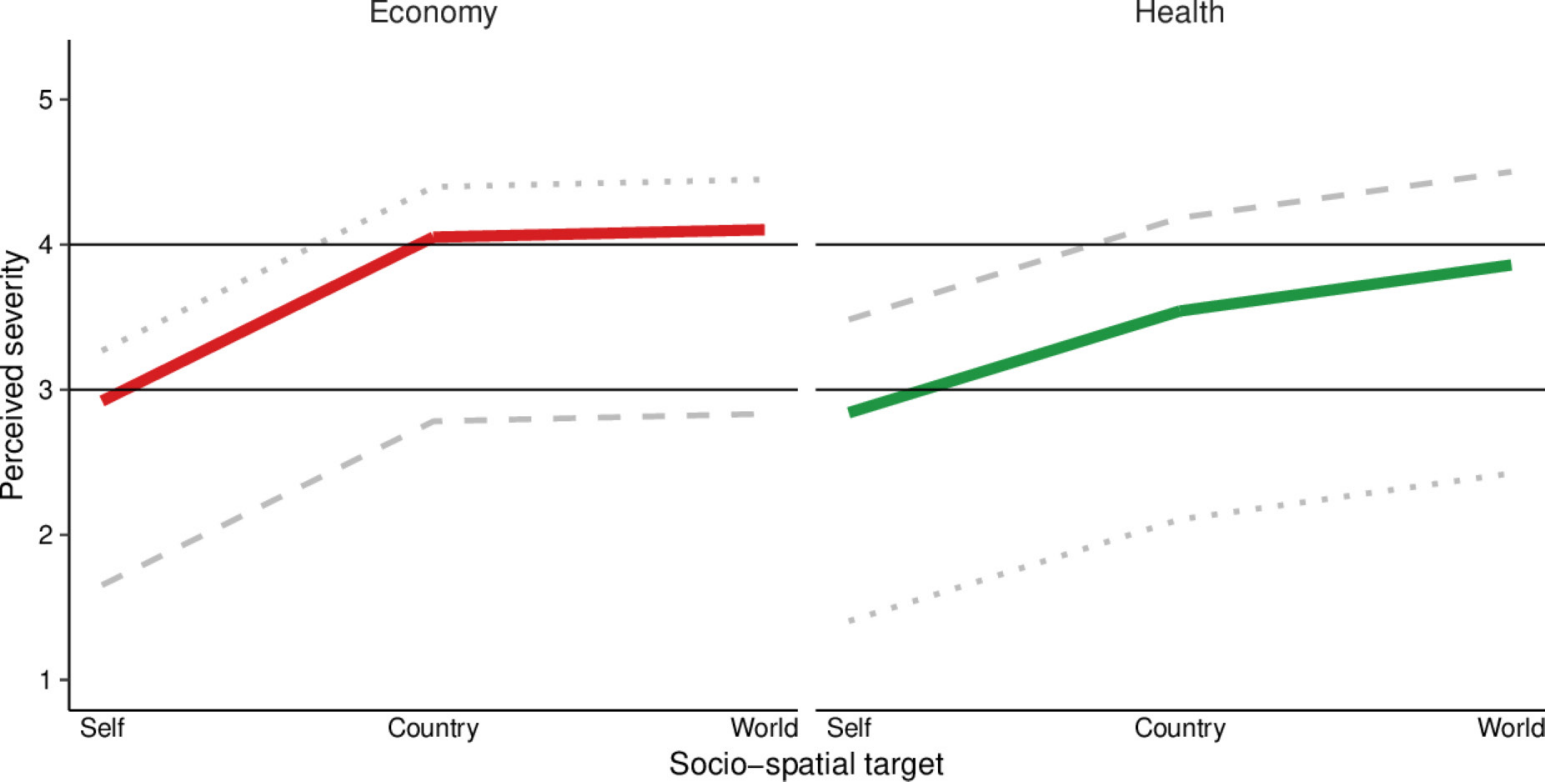
Model-predicted perceived severity of health and economic impacts by socio-spatial target. Colored lines represent the average predicted difference in ratings of economic (red) and health impacts (green) between socio-spatial targets (self, country, world) on a scale from 1−5. Gray lines represent the predicted impact for the smallest (dotted) and largest (dashed) random slope for the domain (reference group: economy). Black lines aim to facilitate comparisons between domains.

When contrasting health and economic impacts, multilevel analyses revealed that participants, on average, rated the economic impact of the COVID-19 pandemic as more serious than the health impact (*b*=−0.08, p<0.001; see figure 4, table 1 and online supplemental table S4). Although this average effect is small, it only refers to the difference between health and economic impacts at the personal level, with the health impact being perceived as less serious by 0.08 scale points on a scale from 1 (not at all serious) to 5 (very serious). Notably, this difference between domains is larger at the national (b=−0.43, p<0.001) and global levels (b=−0.16, p<0.001). Furthermore, we observe substantial variation in the magnitude, and even direction, of the domain effect across individuals, with some participants rating health impacts as more serious (random slopes range=−1.86–1.83).

**Table 1:** Results from multilevel models predicting perceived impacts of the COVID-19 pandemic

	Absolute impact	Relative impact
Intercept	2.92***	0.05***
Health (ref: Econ.)	−0.08***	0.27***
Country (ref: Self)*/Self-Country (ref: Country-World)†	1.13***	1.08***
World (ref: Self)*/Self-World (ref: Country-World)†	1.18***	1.13***
Health x Country*/Self-Country†	−0.43***	−0.69***
Health x World*/Self-World†	−0.16***	−0.43***
Pseudo-R²	0.44	0.32

Coefficients are reported from the overall models including random slopes. All coefficients are unstandardised.

***p<0.001, **p<0.01, *p<0.05.

* Absolute impacts refer to perceived severity ratings for each socio-spatial target and domain.

† Relative impacts are used to test for individual and national optimism. They reflect differences in impacts at different socio-spatial levels within the same domain.

**Figure 5:**
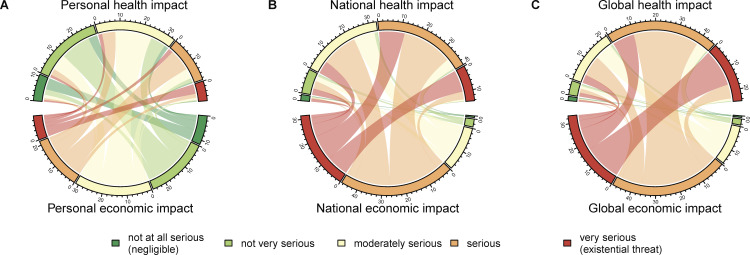
Perceived health and economic impact connections across socio-spatial targets. (A) Personal health impact. (B) National health impact. (C) Global health impact. Percentages of health and economic impact ratings are displayed for each target separately. Connections in between visualize all combinations between ratings.

Participants’ evaluations of health and economic impacts across different socio-spatial targets are represented in a chord diagram (see figure 5). The diagram visualises severity ratings by domain (health vs economic) and by socio-spatial target (self, country, world). Each chord connects individual severity ratings for health and economic impacts within the same socio-spatial target. For instance, the percentage of participants who rated the economic impact as serious is linked to all levels of health impact ratings. Figure 5A shows personal impact ratings, with most chords shaded green, indicating that participants generally perceived the pandemic’s impact on themselves as less severe. Figure 5B represents national-level ratings, with a shift towards red tones, reflecting higher perceived severity. Figure 5C displays global-level ratings, which are predominantly red, indicating that participants viewed the pandemic’s global impact as particularly serious. Overall, the visualisation underscores both the overall trend of increasing perceived severity from personal to global levels and the variability in individual perceptions across domains and spatial levels.

An extended multilevel model including new SARS-CoV-2 cases per million and their interactions with other predictors yielded statistically significant effects. However, the effects were very small in magnitude (all |*b*s| <0.001), and the increase in explained variance was negligible (Δpseudo-*R*^2^ <0.01). These findings on the perceived impact of the COVID-19 pandemic are relatively robust and stable across varying epidemiological conditions.

### Individual and national optimism about the COVID-19 pandemic’s impact

**Figure 6:**
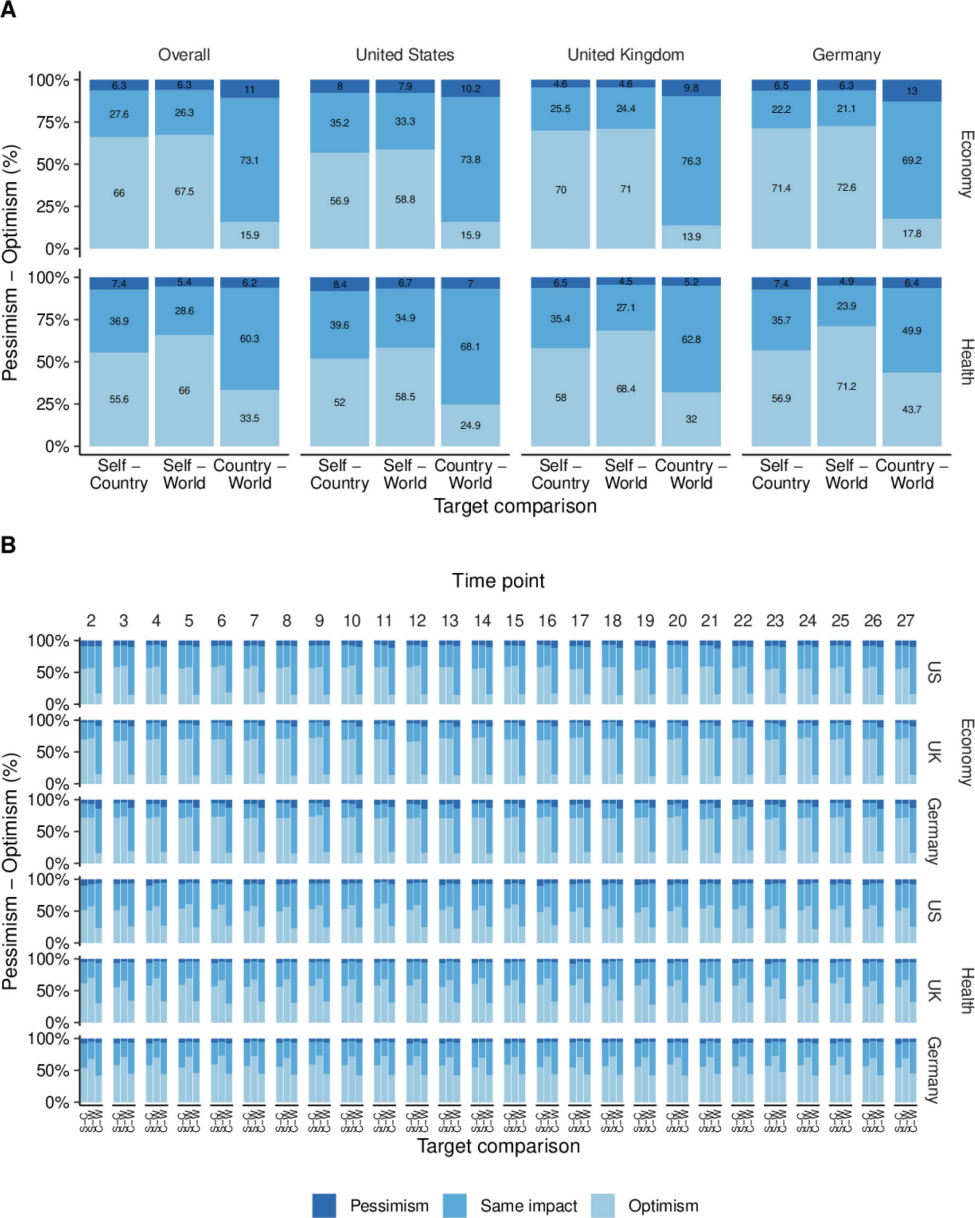
Relative perceived impact by target comparison and domain. Displayed are the proportions of participants who perceived the distant target as worse off (i.e., optimism), both socio-spatial targets as equally affected, or the proximal target as worse off (i.e., pessimism). (A) Proportions by target comparison, domain, and country. (B) Proportions by target comparison, domain, country, and time point.

Most participants exhibited individual optimism regarding the economic impact of the COVID-19 pandemic, rating the impact on themselves as less severe than its impact on national and global economies. Specifically, 66.0% and 67.5% believed they were less economically affected than their national (M=1.13, SD=1.24) and global economies (M=1.18, SD=1.26; see also figure 6A, upper left panel), respectively. In contrast, only 15.9% displayed national optimism, perceiving the national economic impact as less severe than the global economic impact (M=0.05, SD=0.61). The majority of participants (73.1%) believed that national and global economies were equally affected.

Patterns regarding individual and national optimism were consistent across the three different countries, with some differences in magnitude. Most US participants expressed individual optimism about the economic impact of the pandemic, rating their personal economic impact as less severe than the national (56.9%; M=0.85, SD=1.16) and global impacts (58.8%; M=0.91, SD=1.19). UK participants were even more individually optimistic, with the vast majority rating their personal economic impact as less serious than the national (70.0%; M=1.18, SD=1.14) and global economic impacts (71.0%; M=1.22, SD=1.16). Similarly, 71.4% and 72.6% of the German sample viewed their personal economic impact as less severe than the national (M=1.36, SD=1.35) and global economic impacts (M=1.41, SD=1.36).

Across all three countries, national optimism was less common than individual optimism. In the US, only 15.9% of the participants were nationally optimistic, believing that the national economic impact was less severe than the global one. Most US participants viewed the national and global economic impacts as equally serious (73.8%; M=0.06, SD=0.61). Similarly, just 13.9% of the UK participants expressed national optimism, while the majority (76.3%) believed that the national and global economy were equally affected (M=0.04, SD=0.54). Among the German participants, national optimism was somewhat more prevalent (17.8%), though most (69.2%) also rated the national and global impacts as equally serious (M=0.05, SD=0.67).

This general pattern held consistently across the 26 time points in all three countries: individual optimism was always the most common, while individual pessimism was least frequent (see figure 6B). Similarly, most participants consistently viewed the national and global economic impacts as equally serious, followed by those indicating national optimism.

Regarding the health domain, participants also demonstrated individual optimism. A total of 55.6% rated the health impact on themselves as less serious than the national impact (M=0.70, SD=0.99), and 66.0% rated it as less serious than the global impact (M=1.02, SD=1.10; see also figure 6A, lower left panel). National optimism was again less common: only 33.5% believed that the national health impact was less serious than the global impact (M=0.32, SD=0.71). Conversely, the majority (60.3%) rated the national and global health impacts as equally serious.

Similar patterns of individual optimism about health emerged across the three countries. In the US, most participants were individually optimistic, perceiving the health impact on themselves as less severe than on the national (52.0%; M=0.64, SD=0.99) and global level (58.5%; M=0.84, SD=1.07). UK participants showed even greater individual optimism, with 58.0% and 68.4% reporting that their own health was less affected than the national (M=0.73, SD=0.94) and global health (M=1.03, SD=1.03). Similarly, 56.9% and 71.2% of the German participants viewed their personal health impact as less serious than the national (M=0.73, SD=1.04) and global health impacts (M=1.18, SD=1.16).

National optimism about health was less prevalent than individual optimism in all three countries. In the US, only 24.9% of participants believed that the national impact was less severe than the global impact, while the majority (68.1%) viewed both as equally serious (M=0.20, SD=0.63). In the UK, 32.0% of participants expressed national optimism, but 62.8% still rated the national and global health impacts as comparable (M=0.30, SD=0.64). National optimism was most prevalent in Germany (43.7%; M=0.46, SD=0.82), although a slightly larger share (49.9%) rated the national and global health impacts as equally serious.

This pattern remained stable across all 26 time points in each country (see figure 6B): individual optimism about health was consistently the most common response, while individual pessimism was the least frequent. As with the economic domain, the majority of participants rated the national and global health impacts as equally serious, followed by those expressing national optimism.

**Figure 7:**
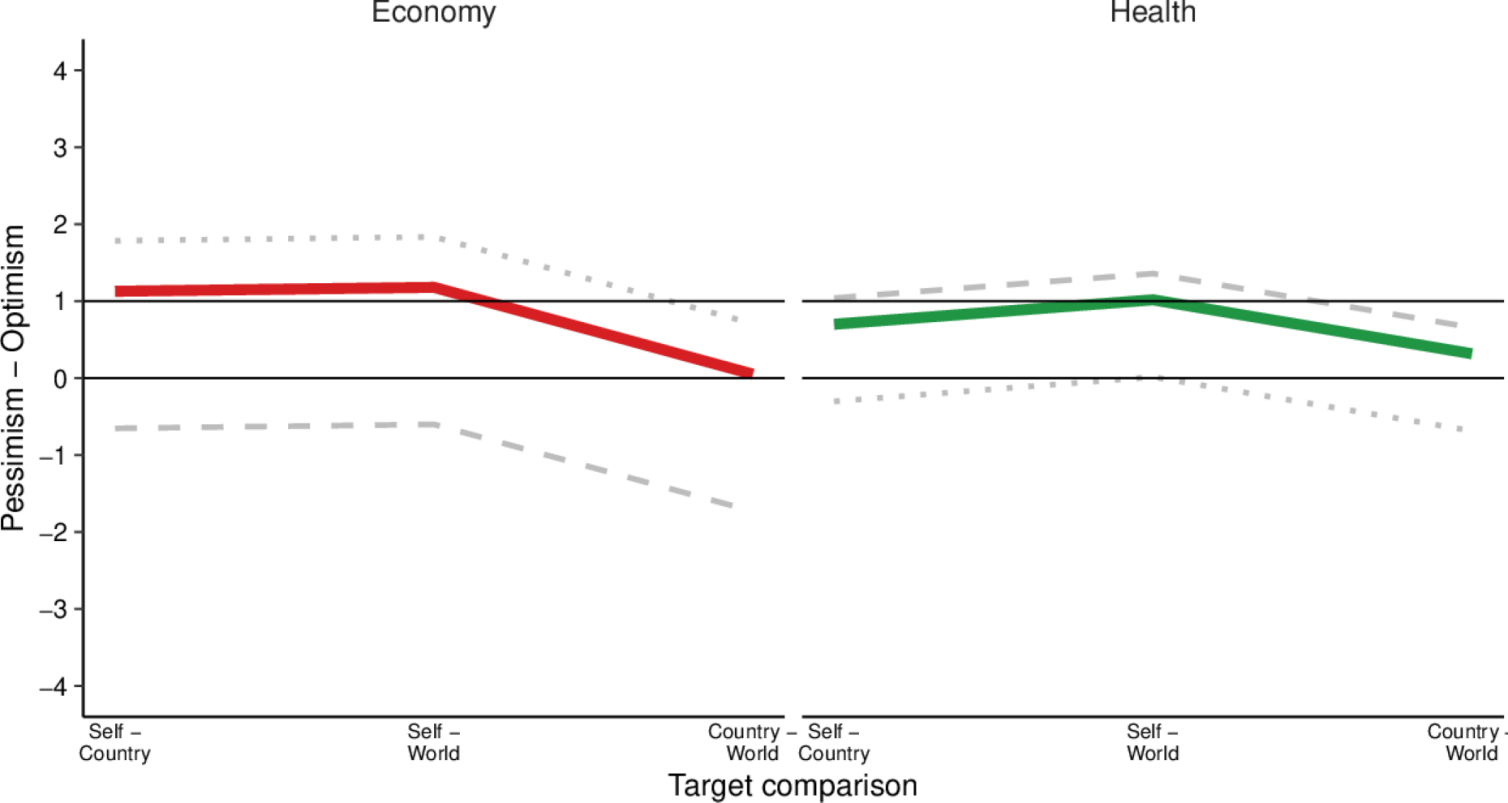
Model-predicted optimism about health and economic impacts. Colored lines represent the average predicted difference in ratings of economic (red) and health impacts (green) between target comparisons (self–country, self–world, country–world) on a scale from −4 to +4. Gray lines represent the predicted impact for the smallest (dotted) and largest (dashed) random slope for the domain (reference group: economy). Black lines aim to facilitate comparisons between domains.

Multilevel analyses further supported the presence of individual optimism, as personal impacts were rated significantly less serious than national (b=1.13, p<0.001) and global impacts (b=1.18, p<0.001; see table 1 and online supplemental table S4). A direct comparison of individual and national optimism showed that national optimism was significantly lower than individual optimism (self–country vs country–world: b=1.08, p<0.001; self–world vs country–world: b=1.13, p<0.001; see figure 7 and online supplemental table S5). Moreover, this difference between individual and national optimism was smaller in the health domain than in the economic domain. Specifically, the interaction effects showed that the gap between self–country and country–world comparisons was smaller for health than for the economy (health (ref.: economy) x self–country (ref.: country–world): b=−0.69, p<0.001; health (ref.: economy) x self–world (ref.: country–world): b=−0.43, p<0.001). As shown in figure 7, this pattern reflects greater individual optimism in the economic domain and relatively greater national optimism in the health domain.

An additional multilevel model including new SARS-CoV-2 cases and their interactions with other predictors yielded statistically significant but negligible effects: all added slopes were very small (all |*b*s|<0.001), and the increase in explained variance was minimal (Δpseudo-*R*^2^<0.01). These results again suggest that observed patterns of optimism are robust across varying epidemiological conditions.

## Discussion

The main aim of the present study was to explore the perceived impact of the COVID-19 pandemic across three countries over a 2-year period. Specifically, we investigated perceived health and economic impacts across different socio-spatial targets (the self, one’s own country, the world). Overall, the impacts were perceived as fairly serious, especially in the economic domain. Yet, participants consistently perceived themselves as less affected than their country or the world, demonstrating individual optimism.^10 16 18 27^ Conversely, only a minority of participants expressed national optimism, perceiving the national impact as less severe than the global impact, particularly in relation to the economy.

Despite considerable variation between countries and pandemic situations,^37^ a consistent pattern emerged across all three national samples: Health impacts were generally rated as less severe than economic impacts, especially on national and global levels. This finding may seem counterintuitive, given that COVID-19 began as a health crisis. However, health and economic impacts are intrinsically linked (e.g., healthcare costs), and participants may have viewed health impacts as contributing to a broader economic burden. Alternatively, this pattern may reflect prevailing stereotypes, particularly in the health domain, which suggested that only certain groups (e.g., older adults) experienced severe or long-lasting health impacts.^27 28 38 39^ Conversely, economic impacts were likely perceived as more widespread, affecting all societal levels regardless of demographic group. Moreover, economic impacts (e.g., job losses) may have been seen as longer lasting or less controllable than health impacts.^27^ For instance, COVID-19 is often associated with relatively mild symptoms (e.g., sore throat, headache) in the short term,^40^ whereas economic recovery may be perceived as a long-term process. In addition, participants may have viewed health impacts as primarily personal issues, while economic impacts were seen as a national responsibility. Regardless of the underlying mechanism, the perception that economic impacts outweighed health impacts may have reduced support for collective health protection measures, due to a perceived trade-off between saving lives and safeguarding livelihoods.^4^

Even in the face of a global crisis, most participants expressed at least individual optimism, rating national and global impacts as more serious than personal impacts. This raises important questions about the origins and accuracy of such perceptions. A pattern of increasing severity ratings at broader socio-spatial scales might be expected if the measures had assessed impacts in absolute terms (e.g., money lost by an individual vs their country), or if participants had been asked to consider specific risks at different levels (e.g., individual infection risk vs healthcare system overload).^20^ However, our measure was relative and designed to be applicable across various reference points (e.g., country size, number of cases). Therefore, it was theoretically possible for participants to perceive themselves as more severely affected than their country. Nonetheless, we consistently observed a pattern of increasing perceived severity from self to world, indicating optimism. While we cannot entirely rule out the possibility of sample bias (e.g., participants who fared relatively well during the pandemic),^12^ this explanation seems unlikely given the large sample size and consistency with previous research.^15 16 18 19^ A more plausible explanation is that individuals were genuinely optimistic to some degree—a perception influenced by factors such as controllability.^15 27 41^ For instance, participants may have believed they had greater capacity to mitigate personal impacts compared to others.^27 41 42^ Furthermore, the abstractness of social–spatial targets may have facilitated downward social comparisons, allowing participants to construe these broader groups as worse off.^28 42^ This cognitive-motivational strategy may have served as a coping mechanism in the face of a threatening global event.^28 43^

Interestingly, although most participants expressed individual optimism, a majority perceived national and global economic impacts as equally serious. This finding is noteworthy given that our samples were drawn from wealthy countries that are arguably better positioned to cope with the pandemic than the global average.^44^ The perception of parity between national and global economic impacts suggests a potential underestimation of global economic inequality and the uneven distribution of resources for mitigating pandemic consequences.^6 45^ One explanation could be that economic impacts were more salient at the national level than the global level, possibly due to differences in media coverage and perceived relevance.^45 46^ For instance, in Portugal, media coverage initially emphasised national consequences following the first domestic SARS-CoV-2 case, while international reporting often focused on health-related indicators.^47^ As a result, differences in the salience between national and global impacts may have been more pronounced in the economic domain than in the health domain. Alternatively, participants may have engaged in ‘motivated reasoning’,^48^ concluding that their own country was equally or even more economically affected than others.^45^ This could involve exaggerating national hardships or downplaying global ones, possibly to maintain a positive image of one’s country as a self-relevant social group—especially in light of unequal global access to pandemic mitigation resources.^45 49^ Unlike health, economic resources are finite and inherently tied to distributional issues.^45^ By discounting disparities in global economic impact, participants may have been able to focus on domestic challenges, potentially reducing feelings of moral obligation to assist other nations. In turn, this perceived comparability of national and global economic consequences may have weakened global solidarity and reduced the perceived need for international aid and cooperation.^45 49^

### Limitations and future research

While the present study yields important insights into the perceived impact of the COVID-19 pandemic over a 2-year period and across three countries, some limitations need to be acknowledged. First, the study focused exclusively on samples from Western, Educated, Industrialized, Rich, and Democratic (WEIRD) countries.^50^ Specifically, the three high-income countries examined—each with advanced economies and greater-than-average resources to mitigate pandemic impacts—may not reflect the experiences of populations in less affluent or non-WEIRD contexts. Consequently, our findings on perceived impacts may not generalise globally. Future research should aim to extend these findings by including more diverse populations, particularly from non-WEIRD countries.

Second, although the samples were approximately representative of each country’s age and gender distribution, other important demographic and socio-economic variables such as education level^51^ were not examined. For instance, while current employment status was assessed, the study did not collect information on prior job loss or unemployment directly attributable to the pandemic. Including such variables in future studies may offer a more comprehensive understanding of how individuals’ perceptions of pandemic impacts are shaped.

Third, although the study includes three countries, it did not account for regional variation within each country. Differences in local mitigation strategies, healthcare capacity and economic conditions may have significantly influenced perceived impacts. The study did not incorporate macrolevel indicators (e.g., public health infrastructure, fiscal policy responses), nor did it explore the broader policy discourse surrounding the pandemic. Future research could address these limitations by integrating regional data and additional objective indicators to contextualise subjective perceptions more fully.

## Conclusion

The present study offers comprehensive insights into how individuals perceived the health and economic impacts of the COVID-19 pandemic across different socio-spatial targets (the self, one’s own country and the world). These findings have important implications for pandemic preparedness and public support for international aid.

A key concern for future pandemic preparedness is the finding that participants perceived economic impacts as more severe than health impacts, particularly at the national and global levels. This relative prioritisation of economic over health impacts may suggest that public attention to global health challenges, such as pandemics, could wane once domestic economic conditions stabilise. Moreover, this prioritisation could undermine support for collective protection measures if these are perceived as harmful for the economy. These results highlight the need for preparedness strategies that anticipate such responses and develop communication and policy approaches to sustain long-term public backing for collective protective efforts.

While individual optimism remained strong even amid a global crisis, national optimism was far less common. Notably, participants from three high-income countries perceived national and global economic impacts as similarly severe—an unexpected finding given their countries’ relatively strong capacities to manage the crisis. This suggests a potential underestimation of global inequalities in access to mitigation resources, which may have weakened public support for assisting less affluent countries and, in turn, undermined global solidarity. As the COVID-19 pandemic has shown, such neglect can have devastating consequences. For instance, in lower middle and low-income countries, more than 50% of deaths could have been prevented with equitable access to vaccines. This starkly illustrates the human cost of vaccine nationalism and global resource disparity.^52^ From a policy perspective, effectively communicating the importance of international solidarity—particularly in wealthier nations—is essential. Raising public awareness of global inequalities in pandemic preparedness and impact could help build broader support for international aid and cooperation during future global crises.

## Supplementary material

10.1136/bmjph-2024-001095 online supplemental file 1

## Data Availability

Data are available in a public, open access repository.
