# Supplementary material for: Public perceptions of health and economic impacts during COVID-19: Findings from a repeated cross-national survey in the US, UK and Germany (2020–2022)
Source: BMJ Public Health. 2025 Oct 5;3(2):e001095. doi: 10.1136/bmjph-2024-001095 (PMC12506208; doi:10.1136/bmjph-2024-001095)
Supplement: online supplemental file 1 [file bmjph-3-2-s001.docx]

**Supplementary Material**

**Figure S1. Items for health and economic impacts on different socio-spatial targets**


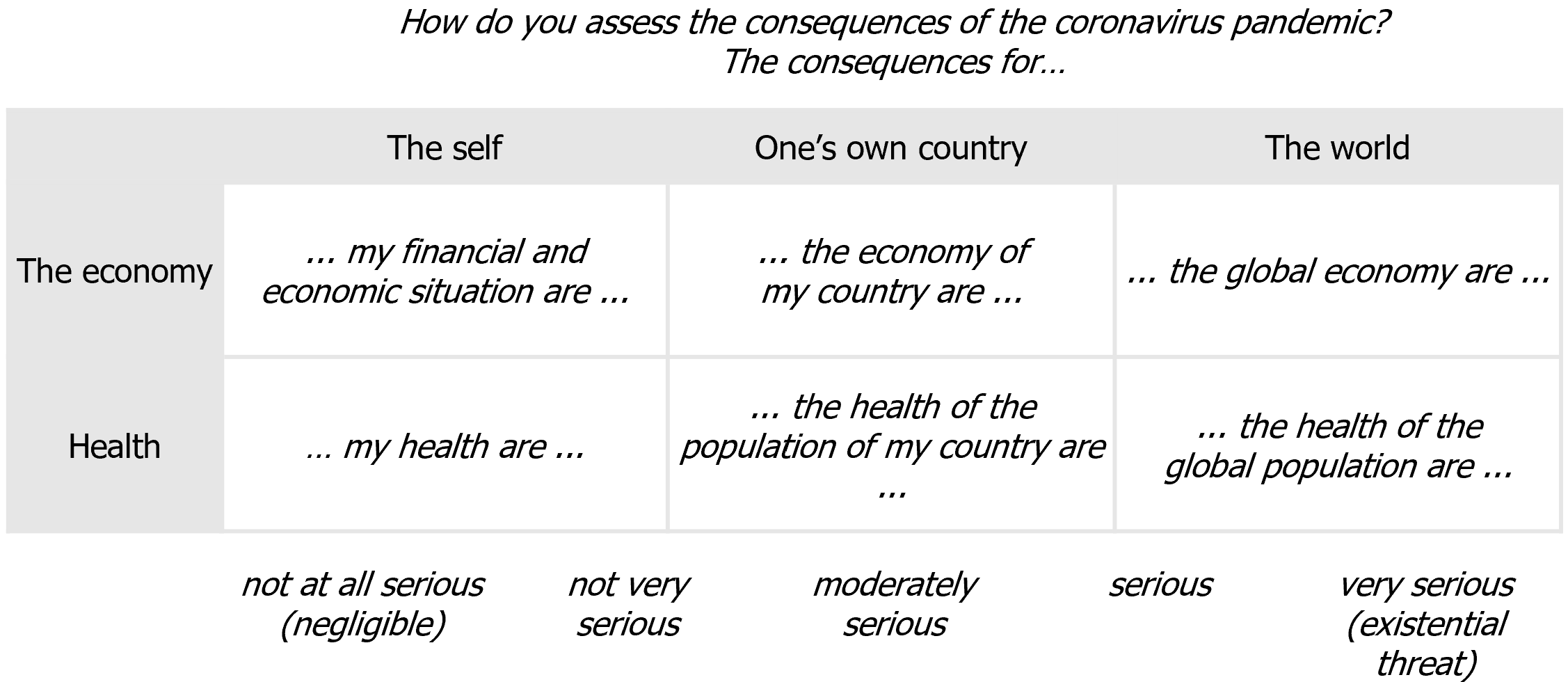


*Note*. German participants received a German version of the items. A version of the questionnaire including these items is published here: Renner B, Koller JE, Villinger K, et al. Questionnaire for the EUCLID project (April 3 - April 16, 2020). KonDATA, July 9, 2025. doi: 10.48606/tywvrp6vrgzhzae9

**Figure S2. Proportion of serious health and economic impacts over time**

**
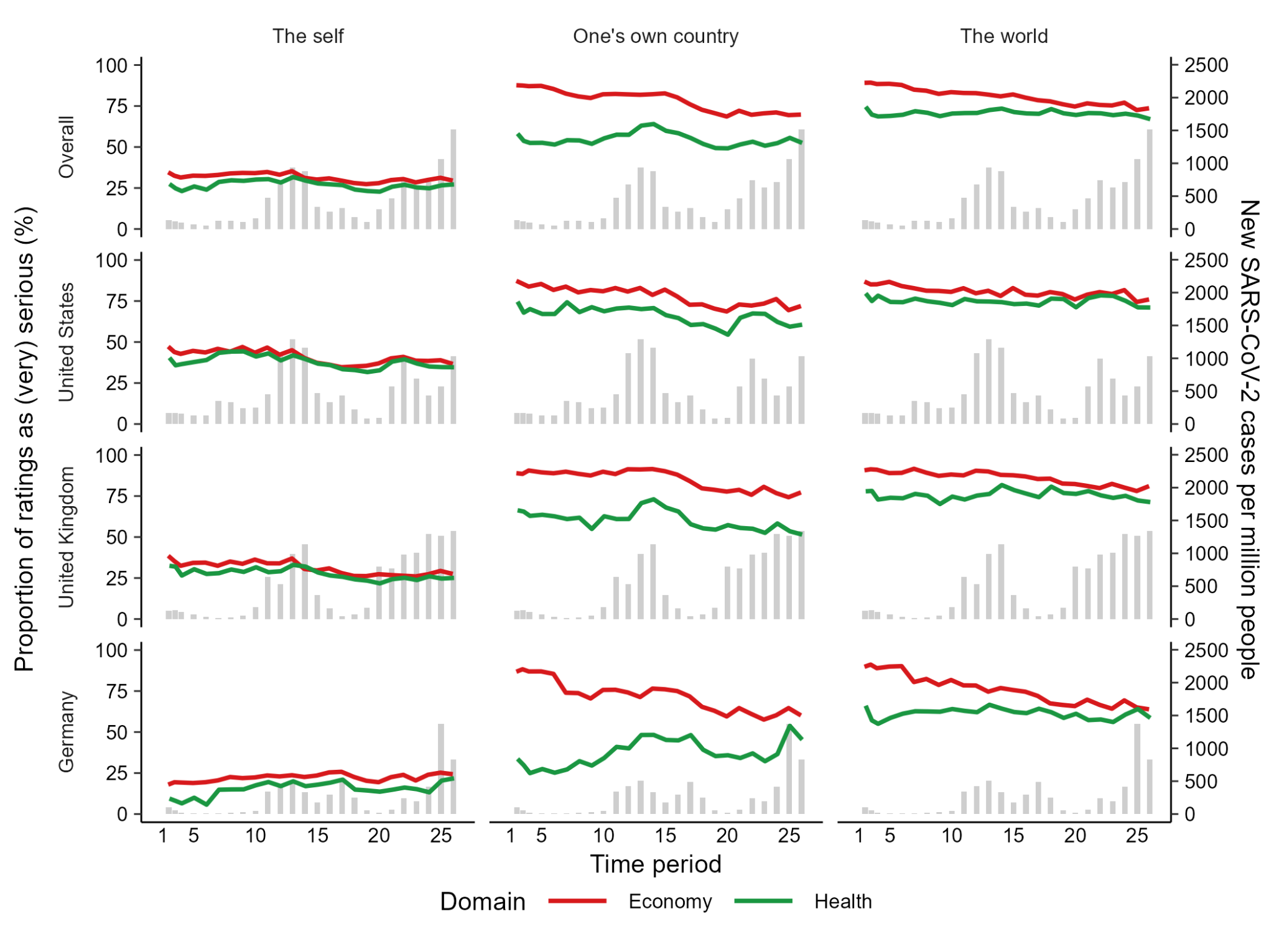
**

*Note*. Colored lines represent the proportion of participants who rated the economic (red) and health impacts (green) as serious or very serious (ratings of 4 and 5 on a scale from 1-5). The x-axis reflects the intervals between waves of data collection (first 4 waves: 2-week intervals; rest: 4-week intervals). The number of daily new SARS-CoV-2 cases per million people (7-day smoothed) is averaged for each time point and is displayed in the background (gray; right y-axis).

**Table S1**

Sample characteristics for the United States

| Sample | Time point | | Sample size | Mean Age (SD) | Women (%) | (Very) Healthy (%) | Income (%) | (Self-) Employed (%) |
| --- | --- | --- | --- | --- | --- | --- | --- | --- |
|  | Start | End |  |  |  |  |  |  |
| Overall | 04/04/20 | 28/01/22 | 26,332 | 45.26 (16.12) | 50.8 | 68.6 | 41.6 | 64.3 |
| 1 | 04/04/20 | 07/04/20 | 994 | 45.46 (15.84) | 51.4 | 67.5 | 44.9 | 64.3 |
| 2 | 18/04/20 | 20/04/20 | 992 | 44.99 (16.19) | 49.9 | 70.4 | 45.7 | 60.9 |
| 3 | 06/05/20 | 08/05/20 | 991 | 44.84 (16.14) | 51.6 | 70.1 | 42.6 | 61.2 |
| 4 | 16/05/20 | 19/05/20 | 994 | 45.28 (16.07) | 51.6 | 67.8 | 45.2 | 62.8 |
| 5 | 13/06/20 | 15/06/20 | 985 | 45.30 (16.34) | 51.7 | 70.0 | 43.7 | 65.3 |
| 6 | 11/07/20 | 13/07/20 | 1,002 | 44.92 (16.19) | 50.7 | 69.2 | 43.8 | 64.0 |
| 7 | 08/08/20 | 14/08/20 | 989 | 45.25 (16.31) | 51.0 | 71.6 | 42.7 | 69.2 |
| 8 | 05/09/20 | 16/09/20 | 1,012 | 45.67 (16.36) | 51.0 | 65.7 | 42.8 | 61.1 |
| 9 | 30/09/20 | 05/10/20 | 1,021 | 45.43 (16.10) | 51.3 | 66.1 | 45.4 | 59.7 |
| 10 | 28/10/20 | 30/10/20 | 1,021 | 45.53 (16.26) | 51.0 | 65.1 | 43.6 | 60.7 |
| 11 | 25/11/20 | 27/11/20 | 1,021 | 45.57 (16.07) | 50.4 | 69.6 | 43.3 | 64.1 |
| 12 | 23/12/20 | 29/12/20 | 1,017 | 45.31 (16.20) | 50.6 | 66.0 | 44.2 | 65.9 |
| 13 | 20/01/21 | 29/01/21 | 1,020 | 45.47 (16.09) | 50.1 | 67.5 | 42.4 | 64.4 |
| 14 | 17/02/21 | 24/02/21 | 1,021 | 46.48 (15.93) | 50.6 | 67.3 | 42.2 | 65.4 |
| 15 | 17/03/21 | 23/03/21 | 1,021 | 46.30 (16.05) | 50.9 | 67.3 | 40.0 | 62.1 |
| 16 | 14/04/21 | 21/04/21 | 1,020 | 46.09 (16.17) | 50.6 | 70.5 | 37.3 | 66.5 |
| 17 | 12/05/21 | 18/05/21 | 1,024 | 46.57 (16.10) | 50.6 | 68.8 | 38.6 | 65.8 |
| 18 | 09/06/21 | 21/06/21 | 1,018 | 46.38 (15.98) | 50.7 | 65.2 | 40.5 | 61.6 |
| 19 | 07/07/21 | 13/07/21 | 1,016 | 45.02 (16.05) | 50.8 | 68.1 | 43.7 | 65.5 |
| 20 | 04/08/21 | 11/08/21 | 1,019 | 44.53 (16.52) | 50.6 | 74.5 | 36.0 | 65.2 |
| 21 | 01/09/21 | 07/09/21 | 1,021 | 44.59 (16.23) | 51.0 | 75.5 | 36.8 | 66.0 |
| 22 | 29/09/21 | 05/10/21 | 1,028 | 44.72 (16.16) | 49.8 | 73.2 | 35.2 | 65.2 |
| 23 | 27/10/21 | 02/11/21 | 1,020 | 45.00 (16.27) | 51.2 | 69.8 | 40.6 | 64.5 |
| 24 | 24/11/21 | 27/11/21 | 1,021 | 41.07 (14.42) | 50.3 | 64.7 | 41.5 | 69.6 |
| 25 | 22/12/21 | 12/01/22 | 1,021 | 45.58 (16.23) | 50.1 | 65.0 | 40.2 | 63.9 |
| 26 | 19/01/22 | 28/01/22 | 1,023 | 45.49 (16.11) | 50.6 | 65.9 | 39.5 | 65.6 |

*Note*. Age is provided in years. The percentage of participants with an income up to 3,000 in the country’s respective currency is reported. Health status was assessed subjectively.

**Table S2**

Sample characteristics for the United Kingdom

| Sample | Time point | | Sample size | Mean Age (SD) | Women (%) | (Very) Healthy (%) | Income (%) | (Self-) Employed (%) |
| --- | --- | --- | --- | --- | --- | --- | --- | --- |
|  | Start | End |  |  |  |  |  |  |
| Overall | 04/04/20 | 25/01/22 | 26,003 | 45.71 (15.62) | 51.3 | 70.8 | 62.6 | 63.7 |
| 1 | 04/04/20 | 07/04/20 | 995 | 45.88 (15.47) | 51.9 | 71.9 | 68.2 | 64.8 |
| 2 | 18/04/20 | 20/04/20 | 1,002 | 45.89 (15.66) | 51.4 | 70.2 | 65.1 | 63.5 |
| 3 | 06/05/20 | 08/05/20 | 989 | 45.78 (15.71) | 51.6 | 74.1 | 62.2 | 62.2 |
| 4 | 19/05/20 | 21/05/20 | 991 | 45.94 (15.75) | 51.2 | 72.7 | 65.8 | 61.1 |
| 5 | 13/06/20 | 15/06/20 | 999 | 46.22 (15.52) | 51.0 | 70.4 | 61.9 | 65.3 |
| 6 | 13/07/20 | 15/07/20 | 1,007 | 46.26 (15.78) | 51.0 | 70.6 | 62.7 | 66.6 |
| 7 | 08/08/20 | 14/08/20 | 1,005 | 46.14 (15.64) | 51.4 | 68.8 | 63.2 | 63.6 |
| 8 | 05/09/20 | 08/09/20 | 1,003 | 46.05 (15.80) | 51.6 | 70.7 | 63.5 | 62.5 |
| 9 | 30/09/20 | 02/10/20 | 1,005 | 46.19 (15.82) | 51.5 | 66.6 | 67.6 | 61.4 |
| 10 | 28/10/20 | 31/10/20 | 1,002 | 45.78 (15.72) | 50.8 | 73.2 | 66.8 | 58.1 |
| 11 | 25/11/20 | 28/11/20 | 1,006 | 45.78 (15.54) | 51.6 | 69.5 | 64.5 | 59.5 |
| 12 | 23/12/20 | 28/12/20 | 1,004 | 46.15 (15.75) | 51.6 | 70.2 | 63.0 | 62.8 |
| 13 | 20/01/21 | 27/01/21 | 997 | 44.18 (16.47) | 51.2 | 73.6 | 60.3 | 62.1 |
| 14 | 17/02/21 | 26/02/21 | 999 | 46.67 (15.50) | 51.0 | 70.1 | 67.0 | 59.7 |
| 15 | 17/03/21 | 23/03/21 | 1,006 | 46.81 (15.65) | 51.2 | 70.0 | 62.0 | 61.7 |
| 16 | 14/04/21 | 16/04/21 | 997 | 47.08 (15.41) | 51.3 | 71.1 | 62.6 | 63.5 |
| 17 | 12/05/21 | 18/05/21 | 1,002 | 46.78 (15.44) | 51.4 | 70.5 | 61.8 | 65.7 |
| 18 | 09/06/21 | 15/06/21 | 997 | 47.07 (15.67) | 51.4 | 72.1 | 62.7 | 61.8 |
| 19 | 07/07/21 | 09/07/21 | 997 | 45.36 (15.66) | 51.0 | 71.7 | 58.8 | 60.9 |
| 20 | 04/08/21 | 10/08/21 | 1,004 | 44.73 (15.60) | 50.6 | 69.6 | 62.3 | 63.4 |
| 21 | 01/09/21 | 07/09/21 | 999 | 44.94 (15.50) | 51.0 | 71.2 | 60.5 | 64.5 |
| 22 | 29/09/21 | 04/10/21 | 997 | 45.16 (15.35) | 51.7 | 71.9 | 58.7 | 66.7 |
| 23 | 27/10/21 | 02/11/21 | 997 | 45.06 (15.67) | 51.3 | 70.2 | 59.0 | 63.8 |
| 24 | 24/11/21 | 29/11/21 | 1,003 | 41.02 (13.69) | 51.0 | 70.7 | 58.9 | 71.0 |
| 25 | 22/12/21 | 12/01/22 | 1,001 | 45.73 (15.69) | 51.6 | 70.6 | 57.8 | 70.6 |
| 26 | 19/01/22 | 25/01/22 | 999 | 45.79 (15.67) | 51.4 | 69.0 | 60.5 | 68.2 |

*Note*. Age is provided in years. The percentage of participants with an income up to 3,000 in the country’s respective currency is reported. Health status was assessed subjectively.

**Table S3**

Sample characteristics for the Germany

| Sample | Time point | | Sample size | Mean Age (SD) | Women (%) | (Very) Healthy (%) | Income (%) | (Self-) Employed (%) |
| --- | --- | --- | --- | --- | --- | --- | --- | --- |
|  | Start | End |  |  |  |  |  |  |
| Overall | 03/04/20 | 24/01/22 | 26,163 | 48.43 (15.63) | 51.1 | 61.8 | 65.8 | 57.3 |
| 1 | 03/04/20 | 16/04/20 | 997 | 47.81 (15.09) | 51.1 | 76.9 | 53.9 | 66.8 |
| 2 | 17/04/20 | 29/04/20 | 1,078 | 47.82 (15.08) | 51.0 | 75.6 | 58.3 | 63.7 |
| 3 | 29/04/20 | 13/05/20 | 1,006 | 47.91 (15.23) | 50.8 | 77.2 | 61.5 | 63.0 |
| 4 | 13/05/20 | 27/05/20 | 1,009 | 48.16 (15.55) | 51.0 | 71.8 | 61.2 | 59.5 |
| 5 | 08/06/20 | 21/06/20 | 1,008 | 48.59 (15.76) | 50.9 | 72.4 | 62.0 | 59.8 |
| 6 | 10/07/20 | 15/07/20 | 1,000 | 48.73 (16.31) | 51.0 | 61.9 | 68.5 | 54.5 |
| 7 | 07/08/20 | 14/08/20 | 1,005 | 48.74 (15.94) | 51.2 | 59.0 | 68.3 | 55.1 |
| 8 | 04/09/20 | 16/09/20 | 1,006 | 48.64 (16.07) | 51.2 | 58.3 | 70.5 | 53.5 |
| 9 | 30/09/20 | 08/10/20 | 1,004 | 48.63 (16.27) | 51.0 | 61.3 | 67.2 | 57.1 |
| 10 | 28/10/20 | 06/11/20 | 1,004 | 48.49 (15.90) | 51.2 | 61.1 | 66.5 | 55.5 |
| 11 | 25/11/20 | 02/12/20 | 1,001 | 48.62 (16.19) | 51.1 | 58.9 | 64.2 | 55.4 |
| 12 | 23/12/20 | 31/12/20 | 1,001 | 48.72 (16.10) | 51.1 | 59.8 | 67.5 | 52.3 |
| 13 | 20/01/21 | 27/01/21 | 1,003 | 48.62 (16.01) | 51.1 | 57.7 | 68.3 | 53.8 |
| 14 | 17/02/21 | 26/02/21 | 1,005 | 47.93 (15.11) | 51.2 | 58.9 | 67.4 | 57.7 |
| 15 | 17/03/21 | 24/03/21 | 1,002 | 48.16 (15.37) | 51.1 | 57.1 | 66.8 | 57.5 |
| 16 | 14/04/21 | 22/04/21 | 1,003 | 48.06 (15.13) | 51.1 | 57.1 | 67.9 | 54.0 |
| 17 | 12/05/21 | 23/05/21 | 1,001 | 48.22 (15.05) | 51.1 | 57.1 | 67.0 | 57.7 |
| 18 | 09/06/21 | 19/06/21 | 1,008 | 48.66 (15.80) | 51.5 | 59.5 | 68.7 | 53.6 |
| 19 | 07/07/21 | 16/07/21 | 1,004 | 48.92 (16.19) | 51.3 | 61.1 | 67.6 | 54.2 |
| 20 | 04/08/21 | 12/08/21 | 1,004 | 47.99 (14.84) | 51.2 | 57.4 | 68.4 | 59.1 |
| 21 | 01/09/21 | 08/09/21 | 1,000 | 48.04 (14.95) | 51.1 | 56.7 | 67.8 | 57.3 |
| 22 | 29/09/21 | 06/10/21 | 1,003 | 49.05 (16.23) | 51.1 | 55.7 | 67.1 | 54.3 |
| 23 | 27/10/21 | 04/11/21 | 1,007 | 48.88 (15.93) | 51.1 | 59.5 | 64.8 | 56.6 |
| 24 | 24/11/21 | 02/12/21 | 999 | 49.06 (16.26) | 51.3 | 57.6 | 65.6 | 57.9 |
| 25 | 22/12/21 | 29/12/21 | 1,005 | 48.47 (15.01) | 50.9 | 58.7 | 64.1 | 61.8 |
| 26 | 19/01/22 | 24/01/22 | 1,000 | 48.25 (14.89) | 51.1 | 56.9 | 68.0 | 57.8 |

*Note*. Age is provided in years. The percentage of participants with an income up to 3,000 in the country’s respective currency is reported. Health status was assessed subjectively.

**Table S4**

Results of the multilevel models to analyze ratings of the absolute pandemic impact

|  | Random slopes model (fixed effects) | | | | | Random intercept model (fixed effects) | | | | |
| --- | --- | --- | --- | --- | --- | --- | --- | --- | --- | --- |
| Predictor | *b [95% CI]* | SE | *t* | df | *p* | *b [95% CI]* | SE | *t* | df | *p* |
| *Overall* |  |  |  |  |  |  |  |  |  |  |
| Intercept | 2.92  [2.92;2.93] | 0.003 | 893.61 | 200659 | < .001 | 2.92  [2.92;2.93] | 0.003 | 848.91 | 295228 | < .001 |
| Health | -0.08  [-0.09;-0.07] | 0.004 | -19.55 | 258023 | < .001 | -0.08  [-0.09;-0.07] | 0.004 | -20.56 | 391522 | < .001 |
| Country | 1.13  [1.12;1.14] | 0.004 | 311.60 | 313173 | < .001 | 1.13  [1.12;1.14] | 0.004 | 286.51 | 391451 | < .001 |
| World | 1.18  [1.17;1.19] | 0.004 | 325.67 | 313265 | < .001 | 1.18  [1.17;1.19] | 0.004 | 299.47 | 391479 | < .001 |
| Health x Country | -0.43  [-0.44;-0.42] | 0.005 | -83.48 | 313214 | < .001 | -0.43  [-0.44;-0.42] | 0.006 | -76.75 | 391470 | < .001 |
| Health x World | -0.16  [-0.17;-0.15] | 0.005 | -31.58 | 313269 | < .001 | -0.16  [-0.17;-0.15] | 0.006 | -29.03 | 391489 | < .001 |
| *United States* |  |  |  |  |  |  |  |  |  |  |
| Intercept | 3.19 [3.18;3.20] | 0.006 | 567.34 | 61917 | < .001 | 3.19  [3.18;3.20] | 0.006 | 539.17 | 88415 | < .001 |
| Health | -0.07 [-0.08;-0.06] | 0.007 | -10.03 | 86214 | < .001 | -0.07  [-0.08;-0.06] | 0.007 | -10.55 | 131289 | < .001 |
| Country | 0.85 [0.84;0.86] | 0.006 | 142.25 | 105005 | < .001 | 0.85  [0.84;0.86] | 0.007 | 130.65 | 131262 | < .001 |
| World | 0.91 [0.90;0.92] | 0.006 | 152.42 | 105037 | < .001 | 0.91  [0.90;0.92] | 0.007 | 139.99 | 131270 | < .001 |
| Health x Country | -0.21 [-0.22;-0.19] | 0.008 | -24.29 | 105030 | < .001 | -0.21  [-0.22;-0.19] | 0.009 | -22.30 | 131272 | < .001 |
| Health x World | -0.07 [-0.08;-0.05] | 0.008 | -8.03 | 105048 | < .001 | -0.07  [-0.09;-0.05] | 0.009 | -7.37 | 131277 | < .001 |
| *United Kingdom* |  |  |  |  |  |  |  |  |  |  |
| Intercept | 2.97  [2.96;2.98] | 0.005 | 578.20 | 70151 | < .001 | 2.97  [2.96;2.98] | 0.005 | 547.46 | 99210 | < .001 |
| Health | -0.05  [-0.07;-0.04] | 0.006 | -8.17 | 91154 | < .001 | -0.05  [-0.07;-0.04] | 0.006 | -8.51 | 129767 | < .001 |
| Country | 1.18  [1.17;1.19] | 0.006 | 202.01 | 103801 | < .001 | 1.18  [1.17;1.19] | 0.006 | 188.98 | 129745 | < .001 |
| World | 1.22  [1.21;1.23] | 0.006 | 209.03 | 103835 | < .001 | 1.22  [1.21;1.23] | 0.006 | 195.56 | 129755 | < .001 |
| Health x Country | -0.45  [-0.46;-0.43] | 0.008 | -54.31 | 103814 | < .001 | -0.45  [-0.47;-0.43] | 0.009 | -50.81 | 129751 | < .001 |
| Health x World | -0.19  [-0.21;-0.17] | 0.008 | -23.13 | 103837 | < .001 | -0.19  [-0.21;-0.17] | 0.009 | -21.64 | 129759 | < .001 |
|  |  |  |  |  |  |  |  |  |  |  |
|  |  |  |  |  |  |  |  |  |  |  |
|  |  |  |  |  |  |  |  |  |  |  |
| *Germany* |  |  |  |  |  |  |  |  |  |  |
| Intercept | 2.60  [2.59;2.61] | 0.006 | 434.96 | 70225 | < .001 | 2.60  [2.59;2.61] | 0.006 | 422.65 | 116550 | < .001 |
| Health | -0.12  [-0.14;-0.11] | 0.008 | -15.24 | 82081 | < .001 | -0.12  [-0.14;-0.11] | 0.007 | -16.15 | 130472 | < .001 |
| Country | 1.36  [1.35;1.37] | 0.007 | 200.41 | 104370 | < .001 | 1.36  [1.35;1.37] | 0.007 | 181.91 | 130450 | < .001 |
| World | 1.41  [1.40;1.42] | 0.007 | 207.93 | 104396 | < .001 | 1.41  [1.40;1.43] | 0.007 | 188.75 | 130461 | < .001 |
| Health x Country | -0.63  [-0.65;-0.61] | 0.010 | -65.84 | 104377 | < .001 | -0.63  [-0.65;-0.61] | 0.011 | -59.74 | 130450 | < .001 |
| Health x World | -0.23 [-0.25;-0.21] | 0.010 | -23.74 | 104392 | < .001 | -0.23 [-0.25;-0.21] | 0.011 | -21.54 | 130459 | < .001 |

*Note*. Random slopes models were preferred when comparing pseudo-*R*^2^ between models with and without random slope for domain and when using likelihood ratio testing. Pseudo-*R*^2^_Overall_=0.44 for the overall model with random slopes. ICC_Overall_=0.23. For health, the reference group is the economy. For target (country, world), the reference group is the self.

**Table S5**

Results of the multilevel models to analyze ratings of the relative pandemic impact

|  | Random slopes model (fixed effects) | | | | | Random intercept model (fixed effects) | | | | |
| --- | --- | --- | --- | --- | --- | --- | --- | --- | --- | --- |
| Predictor | *b [95% CI]* | SE | *t* | df | *p* | *b [95% CI]* | SE | *t* | df | *p* |
| *Overall* |  |  |  |  |  |  |  |  |  |  |
| Intercept | 0.05  [0.04;0.06] | 0.004 | 13.68 | 181591 | < .001 | 0.05  [0.04;0.06] | 0.004 | 14.20 | 307937 | < .001 |
| Health | 0.27  [0.26;0.27] | 0.004 | 60.85 | 281048 | < .001 | 0.27  [0.26;0.27] | 0.004 | 63.10 | 390549 | < .001 |
| Self–Country | 1.08  [1.07;1.08] | 0.004 | 271.40 | 312679 | < .001 | 1.08  [1.07;1.09] | 0.004 | 255.73 | 390519 | < .001 |
| Self–World | 1.13  [1.12;1.14] | 0.004 | 284.30 | 312514 | < .001 | 1.13  [1.12;1.14] | 0.004 | 267.86 | 390475 | < .001 |
| Health x Self–Country | -0.69  [-0.70;-0.68] | 0.006 | -123.57 | 312762 | < .001 | -0.69  [-0.71;-0.68] | 0.006 | -116.44 | 390532 | < .001 |
| Health x Self–World | -0.43  [-0.44;-0.42] | 0.006 | -76.17 | 312642 | < .001 | -0.43  [-0.44;-0.42] | 0.006 | -71.77 | 390497 | < .001 |
| *United States* |  |  |  |  |  |  |  |  |  |  |
| Intercept | 0.06  [0.05;0.07] | 0.006 | 9.93 | 61530 | < .001 | 0.06  [0.05;0.07] | 0.006 | 10.21 | 101892 | < .001 |
| Health | 0.14  [0.12;0.15] | 0.007 | 19.14 | 94881 | < .001 | 0.14  [0.12;0.15] | 0.007 | 19.84 | 130913 | < .001 |
| Self–Country | 0.79  [0.78;0.80] | 0.007 | 120.84 | 104800 | < .001 | 0.79  [0.78;0.80] | 0.007 | 114.06 | 130898 | < .001 |
| Self–World | 0.85  [0.84;0.86] | 0.007 | 130.18 | 104742 | < .001 | 0.85  [0.84;0.86] | 0.007 | 122.88 | 130882 | < .001 |
| Health x Self–Country | -0.34  [-0.36;-0.32] | 0.009 | -37.03 | 104848 | < .001 | -0.34  [-0.36;-0.32] | 0.010 | -34.95 | 130908 | < .001 |
| Health x Self–World | -0.21  [-0.22;-0.19] | 0.009 | -22.21 | 104811 | < .001 | -0.21  [-0.22;-0.19] | 0.010 | -20.96 | 130897 | < .001 |
| *United Kingdom* |  |  |  |  |  |  |  |  |  |  |
| Intercept | 0.04  [0.03;0.05] | 0.006 | 6.87 | 59670 | < .001 | 0.04  [0.03;0.05] | 0.006 | 7.11 | 102101 | < .001 |
| Health | 0.26  [0.24;0.27] | 0.007 | 36.38 | 90388 | < .001 | 0.26  [0.24;0.27] | 0.007 | 37.89 | 129498 | < .001 |
| Self–Country | 1.14  [1.12;1.15] | 0.006 | 179.30 | 103668 | < .001 | 1.14  [1.12;1.15] | 0.007 | 167.60 | 129492 | < .001 |
| Self–World | 1.18  [1.17;1.19] | 0.006 | 185.74 | 103614 | < .001 | 1.18  [1.16;1.19] | 0.007 | 173.58 | 129478 | < .001 |
| Health x Self–Country | -0.71  [-0.72;-0.69] | 0.009 | -78.68 | 103693 | < .001 | -0.71  [-0.72;-0.69] | 0.010 | -73.56 | 129498 | < .001 |
| Health x Self–World | -0.45  [-0.47;-0.43] | 0.009 | -49.91 | 103651 | < .001 | -0.45  [-0.47;-0.43] | 0.010 | -46.65 | 129486 | < .001 |
| *Germany* |  |  |  |  |  |  |  |  |  |  |
| Intercept | 0.05  [0.04;0.07] | 0.007 | 7.33 | 61487 | < .001 | 0.05  [0.04;0.07] | 0.007 | 7.64 | 106661 | < .001 |
| Health | 0.40  [0.39;0.42] | 0.008 | 48.70 | 93644 | < .001 | 0.40  [0.39;0.42] | 0.008 | 50.50 | 130141 | < .001 |
| Self–Country | 1.31  [1.29;1.32] | 0.008 | 173.52 | 104209 | < .001 | 1.31  [1.29;1.32] | 0.008 | 163.51 | 130132 | < .001 |
| Self–World | 1.36  [1.34;1.37] | 0.008 | 180.37 | 104156 | < .001 | 1.36  [1.34;1.38] | 0.008 | 169.96 | 130117 | < .001 |
| Health x Self–Country | -1.04  [-1.06;-1.01] | 0.011 | -97.17 | 104218 | < .001 | -1.04  [-1.06;-1.01] | 0.011 | -91.56 | 130128 | < .001 |
| Health x Self–World | -0.63  [-0.65;-0.61] | 0.011 | -59.27 | 104178 | < .001 | -0.63  [-0.65;-0.61] | 0.011 | -55.84 | 130116 | < .001 |

*Note*. Random slopes models were preferred when comparing pseudo-*R*^2^ between models with and without random slope for domain and when using likelihood ratio testing. Pseudo-*R*^2^_Overall_=0.32 for the overall model with random slopes. ICC_Overall_=0.25. For health, the reference group is the economy. For target comparison (self-country, self-world), the reference group is country-world.

**Table S6**

Results of the control analyses of the overall multilevel models to analyze ratings of the absolute pandemic impact

|  | Random slopes model (fixed effects) | | | | | Random intercept model (fixed effects) | | | | |
| --- | --- | --- | --- | --- | --- | --- | --- | --- | --- | --- |
| Predictor | *b [95% CI]* | SE | *t* | df | *p* | *b [95% CI]* | SE | *t* | df | *p* |
| *Model controlled for age* | | | | | | | | | | |
| Intercept | 2.92  [2.91;2.94] | 0.007 | 392.22 | 95891 | <.001 | 2.90  [2.89;2.92] | 0.008 | 380.88 | 99083 | <.001 |
| Health | -0.08  [-0.09;-0.07] | 0.004 | -19.55 | 258023 | <.001 | -0.08  [-0.09;-0.07] | 0.004 | -20.56 | 391521 | <.001 |
| Country | 1.13  [1.12;1.14] | 0.004 | 311.60 | 313173 | <.001 | 1.13  [1.12;1.14] | 0.004 | 286.51 | 391451 | <.001 |
| World | 1.18  [1.17;1.19] | 0.004 | 325.67 | 313265 | <.001 | 1.18  [1.17;1.19] | 0.004 | 299.47 | 391479 | <.001 |
| Age | -0.00  [-0.00;0.00] | 0.000 | 0.00 | 78467 | .997 | 0.00  [0.00;0.00] | 0.000 | 3.06 | 78473 | .002 |
| Health x Country | -0.43  [-0.44;-0.42] | 0.005 | -83.48 | 313214 | <.001 | -0.43  [-0.44;-0.42] | 0.006 | -76.75 | 391470 | <.001 |
| Health x World | -0.16  [-0.17;-0.15] | 0.005 | -31.58 | 313269 | <.001 | -0.16  [-0.17;-0.15] | 0.006 | -29.03 | 391489 | <.001 |
| *Model controlled for health* | | | | | | | | | | |
| Intercept | 3.19  [3.17;3.21] | 0.010 | 318.40 | 86653 | <.001 | 3.24  [3.22;3.26] | 0.010 | 317.32 | 88131 | <.001 |
| Health | -0.08  [-0.09;-0.08] | 0.004 | -20.10 | 254200 | <.001 | -0.08  [-0.09;-0.08] | 0.004 | -21.14 | 386476 | <.001 |
| Country | 1.13  [1.12;1.13] | 0.004 | 309.49 | 309136 | <.001 | 1.13  [1.12;1.14] | 0.004 | 284.42 | 386408 | <.001 |
| World | 1.18  [1.17;1.19] | 0.004 | 323.36 | 309228 | <.001 | 1.18  [1.17;1.19] | 0.004 | 297.19 | 386436 | <.001 |
| Subj. health | -0.07  [-0.07;-0.07] | 0.002 | -28.09 | 774444 | <.001 | -0.08  [-0.09;-0.08] | 0.003 | -32.42 | 77450 | <.001 |
| Health x Country | -0.43  [-0.44;-0.42] | 0.005 | -82.57 | 309177 | <.001 | -0.43  [-0.44;-0.41] | 0.006 | -75.87 | 386426 | <.001 |
| Health x World | -0.16  [-0.17;-0.15] | 0.005 | -31.20 | 309232 | <.001 | -0.16  [-0.17;-0.15] | 0.006 | -28.67 | 386445 | <.001 |
| *Model controlled for income* | | | | | | | | | | |
| Intercept | 2.94  [2.93;2.94] | 0.004 | 763.35 | 163907 | <.001 | 2.93  [2.93;2.94] | 0.004 | 731.54 | 200993 | <.001 |
| Health | -0.08  [-0.09;-0.07] | 0.004 | -19.12 | 256624 | <.001 | -0.08  [-0.09;-0.07] | 0.004 | -20.11 | 388628 | <.001 |
| Country | 1.13  [1.12;1.13] | 0.004 | 309.90 | 310854 | <.001 | 1.13  [1.12;1.13] | 0.004 | 285.10 | 388558 | <.001 |
| World | 1.18  [1.17;1.18] | 0.004 | 323.89 | 310946 | <.001 | 1.18  [1.17;1.19] | 0.004 | 298.00 | 388586 | <.001 |
| Income | -0.03  [-0.04;-0.02] | 0.005 | -6.83 | 77878 | <.001 | -0.03  [-0.03;-0.02] | 0.005 | -5.36 | 77884 | <.001 |
| Health x Country | -0.43  [-0.44;-0.42] | 0.005 | -82.98 | 310895 | <.001 | -0.43  [-0.44;-0.42] | 0.006 | -76.32 | 388578 | <.001 |
| Health x World | -0.16  [-0.17;-0.15] | 0.005 | -31.47 | 310950 | <.001 | -0.16  [-0.17;-0.15] | 0.006 | -28.94 | 388597 | <.001 |
| *Model controlled for employment status* | | | | | | | | | | |
| Intercept | 2.93  [2.92;2.93] | 0.004 | 784.41 | 170473 | <.001 | 2.92  [2.91;2.93] | 0.004 | 748.78 | 214117 | <.001 |
| Health | -0.08  [-0.09;-0.07] | 0.004 | -19.31 | 256796 | <.001 | -0.08  [-0.09;-0.07] | 0.004 | -20.30 | 389504 | <.001 |
| Country | 1.13  [1.12;1.14] | 0.004 | 310.87 | 311560 | <.001 | 1.13  [1.12;1.14] | 0.004 | 285.87 | 389434 | <.001 |
| World | 1.18  [1.17;1.19] | 0.004 | 324.90 | 311650 | <.001 | 1.18  [1.17;1.19] | 0.004 | 298.79 | 389461 | <.001 |
| Education/ training | -0.05  [-0.07;-0.04] | 0.009 | -5.75 | 78033 | <.001 | -0.06  [-0.08;-0.04] | 0.009 | -6.03 | 78039 | <.001 |
| Unemployed | 0.12 [0.10;0.14] | 0.008 | 14.36 | 78034 | <.001 | 0.13 [0.11;0.14] | 0.009 | 14.94 | 78052 | <.001 |
| Retired | -0.06 [-0.08;-0.05] | 0.006 | -10.68 | 78074 | <.001 | -0.03 [-0.04;-0.02] | 0.006 | -5.24 | 78080 | <.001 |
| Homemaker | -0.01 [-0.03;0.02] | 0.011 | -0.54 | 78054 | .589 | 0.01 [-0.02;0.03] | 0.011 | 0.46 | 78055 | .646 |
| Health x Country | -0.43  [-0.44;-0.42] | 0.005 | -83.33 | 311600 | <.001 | -0.43  [-0.44;-0.42] | 0.006 | -76.61 | 389453 | <.001 |
| Health x World | -0.16  [-0,17;-0.15] | 0.005 | -31.55 | 311655 | <.001 | -0.16  [-0.17;-0.15] | 0.006 | -29.01 | 389472 | <.001 |

*Note*. Random slopes models were preferred when comparing pseudo-*R*^2^ between models with and without random slope for domain and when using likelihood ratio testing. ICC_Overall_=0.23. For health, the reference group is the economy. For target (country, world), the reference group is the self. For income (more than 3000, the reference group is income up to 3,000. For employment status (education/ training, unemployed, retired, homemaker), the reference group is (self-)employed.

**Table S7**

Results of the control analyses of the overall multilevel models to analyze ratings of the relative pandemic impact

|  | Random slopes model (fixed effects) | | | | | Random intercept model (fixed effects) | | | | |
| --- | --- | --- | --- | --- | --- | --- | --- | --- | --- | --- |
| Predictor | *b [95% CI]* | SE | *t* | df | *p* | *b [95% CI]* | SE | *t* | df | *p* |
| *Model controlled for age* | | | | | | | | | | |
| Intercept | -0.19  [-0.21;-0.18] | 0.008 | -24.49 | 103457 | <.001 | -0.23  [-0.25;-0.22] | 0.008 | -29.97 | 101093 | <.001 |
| Health | 0.27  [0.26;0.27] | 0.004 | 60.85 | 281056 | <.001 | 0.27  [0.26;0.27] | 0.004 | 63.10 | 390557 | <.001 |
| Self–Country | 1.08  [1.07;1.08] | 0.004 | 271.40 | 312683 | <.001 | 1.08  [1.07;1.09] | 0.004 | 255.73 | 390527 | <.001 |
| Self–World | 1.13  [1.12;1.14] | 0.004 | 284.30 | 312518 | <.001 | 1.13  [1.12;1.14] | 0.004 | 267.86 | 390482 | <.001 |
| Age | 0.01  [0.00;0.01] | 0.000 | 35.35 | 78495 | <.001 | 0.01  [0.01;0.01] | 0.000 | 41.17 | 78487 | <.001 |
| Health x Self–Country | -0.69  [-0.70;-0.68] | 0.006 | -123.57 | 312765 | <.001 | -0.69  [-0.71;-0.68] | 0.006 | -116.43 | 390539 | <.001 |
| Health x Self–World | -0.43  [-0.44;-0.42] | 0.006 | -76.17 | 312645 | <.001 | -0.43  [-0.44;-0.42] | 0.006 | -71.77 | 390505 | <.001 |
| *Model controlled for health* | | | | | | | | | | |
| Intercept | -0.39  [-0.41;-0.37] | 0.010 | -37.64 | 91035 | <.001 | -0.37  [-0.40;-0.35] | 0.011 | -35.65 | 89008 | <.001 |
| Health | 0.26  [0.26;0.27] | 0.004 | 60.22 | 277629 | <.001 | 0.26  [0.26;0.27] | 0.004 | 62.45 | 385522 | <.001 |
| Self–Country | 1.08  [1.07;1.08] | 0.004 | 269.61 | 308658 | <.001 | 1.08  [1.07;1.08] | 0.004 | 254.09 | 385494 | <.001 |
| Self–World | 1.13  [1.12;1.13) | 0.004 | 282.32 | 308492 | <.001 | 1.13  [1.12;1.14] | 0.004 | 266.05 | 385449 | <.001 |
| Subj. health | 0.12  [0.11;0.12] | 0.003 | 45.64 | 77446 | <.001 | 0.11  [0.11;0.12] | 0.003 | 43.17 | 77442 | <.001 |
| Health x Self–Country | -0.69  [-0.70;-0.68] | 0.006 | -122.23 | 308735 | <.001 | -0.69  [-0.70;-0.68] | 0.006 | -115.19 | 385505 | <.001 |
| Health x Self–World | -0.43  [-0.44;-0.41] | 0.006 | -75.32 | 308617 | <.001 | -0.43  [-0.44;-0.41] | 0.006 | -70.98 | 385471 | <.001 |
| *Model controlled for income* | | | | | | | | | | |
| Intercept | 0.02  [0.01;0.03] | 0.004 | 4.66 | 173307 | <.001 | 0.01  [0.01;0.02] | 0.004 | 3.29 | 210767 | .001 |
| Health | 0.26  [0.26;0.27] | 0.004 | 60.38 | 278978 | <.001 | 0.26  [0.26;0.27] | 0.004 | 62.62 | 387678 | <.001 |
| Self–Country | 1.07  [1.07;1.08] | 0.004 | 269.92 | 310372 | <.001 | 1.07  [1.07;1;08] | 0.004 | 254.34 | 387647 | <.001 |
| Self–World | 1.13  [1.12;1.13] | 0.004 | 282.75 | 310205 | <.001 | 1.13  [1.12;1.13] | 0.004 | 266.40 | 387602 | <.001 |
| Income | 0.07  [0.06;0.08] | 0.005 | 15.09 | 77893 | <.001 | 0.09  [0.08;0.10] | 0.005 | 17.88 | 77886 | <.001 |
| Health x Self–Country | -0.69  [-0.70;-0.68] | 0.006 | -122.77 | 310452 | <.001 | -0.69  [-0.70;-0.68] | 0.006 | -115.68 | 387661 | <.001 |
| Health x Self–World | -0.43  [-0.44;-0.42] | 0.006 | -75.72 | 310332 | <.001 | -0.43  [-0.44;-0.41] | 0.006 | -71.34 | 387627 | <.001 |
| *Model controlled for employment status* | | | | | | | | | | |
| Intercept | 0.01  [0.01;0.02] | 0.004 | 3.37 | 177325 | <.001 | 0.01  [0.00;0.02] | 0.004 | 2.75 | 227667 | .006 |
| Health | 0.27  [0.26;0.27] | 0.004 | 60.70 | 279600 | <.001 | 0.27  [0.26;0.27] | 0.004 | 62.95 | 388540 | <.001 |
| Self–Country | 1.08  [1.07;1.09] | 0.004 | 270.80 | 311069 | <.001 | 1.08  [1.07;1.09] | 0.004 | 255.16 | 388510 | <.001 |
| Self–World | 1.13  [1.12;1.14] | 0.004 | 283.66 | 310906 | <.001 | 1.13  [1.12;1.14] | 0.004 | 267.26 | 388465 | <.001 |
| Education/ training | 0.10  [0.08;0.12] | 0.010 | 10.35 | 78019 | <.001 | 0.08  [0.06;0.10] | 0.010 | 8.27 | 78017 | <.001 |
| Unemployed | -0.15 [-0.16;-0.13] | 0.009 | -16.86 | 78070 | <.001 | -0.17 [-0.19;-0.15] | 0.009 | -19.61 | 78046 | <.001 |
| Retired | 0.23 [0.21;0.24] | 0.006 | 36.31 | 78117 | <.001 | 0.26 [0.24;0.27] | 0.006 | 40.93 | 78106 | <.001 |
| Homemaker | 0.03 [0.01;0.05] | 0.011 | 2.58 | 78051 | .010 | 0.04 [0.02;0.06] | 0.011 | 3.41 | 78047 | <.001 |
| Health x Self–Country | -0.69  [-0.70;-0.68] | 0.006 | -123.32 | 311152 | <.001 | -0.69  [-0.71;-0.68] | 0.006 | -116.20 | 388523 | <.001 |
| Health x Self–World | -0.43  [-0.44;-0.42] | 0.006 | -76.03 | 311033 | <.001 | -0.43  [-0.44;-0.42] | 0.006 | -71.64 | 388488 | <.001 |

*Note*. Random slopes models were preferred when comparing pseudo-*R*^2^ between models with and without random slope for domain and when using likelihood ratio testing. ICC_Overall_=0.25. For health, the reference group is the economy. For target comparison (self-country, self-world), the reference group is country-world. For income (more than 3000, the reference group is income up to 3,000. For income (more than 3000, the reference group is income up to 3,000. For employment status (education/ training, unemployed, retired, homemaker), the reference group is (self-)employed.
